# Comparative transcriptomic analysis reveals differences in *MADS‐box* genes of different hypericum in Changbai Mountains

**DOI:** 10.1002/ece3.10196

**Published:** 2023-06-13

**Authors:** Xia Yunrui, Song Rui, Yang Xing, Zhao Zhe, Zhang Keqin, Zhang Nanyi

**Affiliations:** ^1^ Jilin Provincial Key Laboratory of Tree and Grass Genetics and Breeding, College of Forestry and Grassland Science Jilin Agricultural University Changchun Jilin Province China; ^2^ Jilin Agricultural Science and Technology University Jilin Jilin Province China

**Keywords:** differential gene, divergence time, Hypericum, *Hypericum attenuatum* Choisy, *Hypericum longistylum* Oliv., phylogeny analysis, positive selection

## Abstract

To explore the differences between the hypericum in the Changbai Mountains, we carried out a transcriptome analysis of two common hypericums in the area, which was *Hypericum attenuatum* Choisy and *Hypericum longistylum* Oliv. We screened the *MADS‐box* genes to analyze divergence time and evolutionary selection expression, and determine their expression levels. The results showed that we detected 9287 differentially expressed genes in the two species, of which shared 6044 genes by the two species. Analysis of the selected *MADS* genes revealed that the species was in an environment adapted to its natural evolution. The divergence time estimation showed that the segregation of these genes in the two species was related to the changes of external environment and genome replication events. The results of relative expression showed that the later flowering period of *Hypericum attenuatum* Choisy was related to the higher expression of the *SVP (SHORT VEGETATIVE PHASE)* and the *AGL12 (AGAMOUS LIKE 12)*, while the lower expression of the *FUL (FRUITFULL)*.

## Abstract 2

The results showed that we detected 9287 differentially expressed genes in the two Hypericums, of which shared 6044 genes by the two species. The divergence time analysis showed that the divergence of type II MADS genes between the two species may be related to environmental changes and their own genome replication events. The relative expression analysis showed that the high expression of SVP and AGL12, which inhibited flowering, and the low expression of FUL, which promoted flower development, might be internal factors contributing to the later flowering of *Hypericum attenuatum* than *Hypericum longistylum*.

## 1 INTRODUCTION

Hypericum belongs to *Clusiaceae* Lindl., which are distributed globally. There are nine *Clusiaceae* widely distributed, namely, *Cratoxylum* Blume, *Eliea* Cambess., *Harungana* Lamarck, Hypericum L., *Lianthus* N. Robson, *Santomasia* N. Robson, *Thornea* Breedlove & McClintock, *Triadenum* Rafinesque, and *Vismia* Vand (Crockett & Norman, 2011). The Changbai Mountains (38°46′–47°30′N, 121°08′–134°E) are the highest mountains in the eastern margin of Eurasia (Wang, Jiang, et al., 2022). They are located in northeast China and have a typical temperate continental mountain climate. The main species distributed in the Changbai Mountain area are *Hypericum longistylum* Oliv., and *Hypericum attenuatum* Choisy (Zhou, 2010). Through the previous study, we found that the distribution of these two species in this area has obvious differences: *H. longistylum* is widely distributed and the population density is large; However, the distribution of *H. attenuatum* is narrow and the population density is very small, even reaching the degree of endangered (Li, 2008; Zhang et al., 2018). The difference of habitat and distribution must have the law and mechanism of ecological differentiation. However, research on these two plants has mainly focused on the pharmacology and applications of chemically active ingredients (Li et al., 2012, 2013; Wu et al., 2016). Studies have found that the content of hypericin in the flowers of *H. longistylum* is higher, and it reaches its peak during the flowering period (Zhang et al., 2011). However, Zhang et al. (2018) and Zhang et al. (2011) found that the flowering time of *H. attenuatum* was shorter than that of *H. longistylum*, and the flower size was completely different from that of *H. longistylum*. Therefore, it is of great significance to study the differences between these two hypericum species to make better use of hypericum plants and extract their chemically active ingredients.

The *MADS‐box* gene family is closely related to the flowering stage of flowering plants. This gene family not only plays an important role in controlling the flowering of plants in the five‐round flowering development mechanism but is also related to the regulation of vegetative growth and the regulation process of plant stress resistance (Gramzow & Theissen, 2010; Wang, 2009). The *MADS‐box* gene family is divided into two types, type I and type II (Kerstin et al., 2005; Purugganan et al., 1995). The type I *MADS‐box* gene has an SRF‐like MADS region that can encode a highly conserved protein, which usually contains 1–2 exons (Bodt et al., 2003; Elena et al., 2000; Theissen et al., 1996). The type II *MADS‐box* gene contains an MEF2‐like MADS region with multiple introns and exons. This region usually includes six introns and seven exons, and the MADS region of type II is more conserved than that of type I (Hileman et al., 2006; Kerstin et al., 2005). In addition, the type II *MADS‐box* gene has a semiconserved K domain, which can be used to help the transcription factor protein dimer and DNA combine into a complex domain and a nonconserved C‐terminal domain, which is located between the M region and the K region. Therefore, the type II gene is also called a MIKC‐type gene (Hileman et al., 2006; Ma et al., 1991; Purugganan et al., 1995). According to other research, in angiosperms, type II genes are divided into MIKC^C^‐type and MIKC*‐type subgroups, MIKC^C^‐type *MADS‐box* genes are also divided into 12 subgroups, and these subgroup genes are all related to the ABCDE model of flower organ identity (Chiruta et al., 2010; Favaro et al., 2003; Smaczniak et al., 2012). Among them, the *AGL12* (*AGAMOUS LIKE 12*) subgroup has not only been verified to have regulate the cell cycle of root development but also be a catalyst of the flowering transition (Tapia‐Lopez et al., 2008). The *FUL* (*FRUITFULL*) protein in the SQUA subgroup has multiple functions as a meristem characteristic gene, such as regulating the annual life cycle of plants, promoting fruit development, and promoting stem and leaf growth together with the SOC1 protein (Ferrándiz, Gu, et al., 2000; Ferrándiz, Liljegren, & Yanofsky, 2000; Gu et al., 1998). The *SVP (SHORT VEGETATIVE PHASE)* protein in the STMADS11 subgroup is a repressor protein of flowering transition (Hartmann et al., 2000), which has the function of inhibiting flowering.

With the continuous establishment and improvement of animal and plant genome databases, the use of bioinformatics methods to study the developmental relationship and evolutionary pressure between gene sequences has become increasingly extensive. The nucleotide replacement rate (*ω* = d_*N*_/d_*S*_) is called selection pressure and is the ratio of nonsynonymous (d_*N*_) to synonymous (d_*S*_) substitutions. This ratio can reflect the evolutionary trend of species. The high ratio indicates that the species is in positive selection; The low ratio indicates that it is in purification selection (Dong et al., 2021; Minozzo et al., 2020). By studying these selection pressures, researchers can predict whether a gene has made a new change, and then perform functional validation to determine whether the change has made a new function (Minozzo et al., 2020).

In this study, we selected the leaves of Hypericum plants in the young stage of Changbai Mountain and analyzed their transcriptome data. We screened the transcriptome database, selected the *MADS‐box* gene family among the differentially expressed genes (DEGs), and analyzed its phylogeny and selective evolution to discover the genetic relationship among Hypericum on Changbai Mountain, as well as the selective pressure on plant evolution. This provides theoretical support for the protection and rational utilization of hypericum.

## 2 MATERIALS AND METHODS

### 2.1 Sample collection

The materials used in this study were *H. longistylum* and *H. attenuatum*. All samples were collected in Zuojia Nature Reserve (44°00′–44°07′N, 126°01′–126°08′E), Jilin Province, China. Selected three replicates of each plant, selecting pre‐flowering, young, fully expanded leaves without insect infestation.

### 2.2 Establishment of the transcriptome database of Hypericum

RNA sequencing was based on the HiSeq platform, which sequences all mRNAs transcribed from these two species of Hypericum. The sequencing experiment used the Illumina TruSeqTM RNA Sample Prep Kit method for library construction.

### 2.3 Transcriptome analysis

We performed data quality testing on the original data after sequencing and performed assembly, de‐redundancy, and open reading frame prediction on the data that met the requirements. As a result, we performed an orthologous gene search and gene function annotation. The above work of library construction, sequencing, and transcriptome analysis was entrusted to Shanghai Majorbio Company. The transcriptome data of two hypericum species in the Changbai Mountain area were compared by merging three copies. Taking *H. longistylum*. as the control, if the gene expression level in *H. attenuatum* was significantly greater than that of *H. longistylum*, the gene was considered to be upregulated; otherwise, it was considered to be downregulated.

### 2.4 *MADS‐box* gene family screening and ORF prediction

The transcriptome data were collated and *MADS* genes were screened out (data not provided). Total RNA was extracted by modified Trizol method and reverse transcribed with ReverTra Ace qPCR RT Kit (TOYOBO) (Chang et al., 2014). The differentially expressed *MADS‐box* genes were BLAST aligned in the UniProtKB/SwissProt database on the NCBI website (https://www.ncbi.nlm.nih.gov/), and their ORF (Open Reading Frame) were predicted (https://www.ncbi.nlm.nih.gov/orffinder/).

### 2.5 *MADS‐box* gene domain and motif prediction

The online analysis software MEME (http://meme‐suite.org/) was used to predict the motifs of the selected gene sequences. The motifs of the same type appeared only 0 or once in a sequence, and the maximum number of motifs of a sequence was set as 7. Used NCBI Domains & Structures (https://www.ncbi.nlm.nih.gov/Structure/cdd/wrpsb.cgi) to make predictions about the structure of the selected genes. Visual analysis was performed using TBtools software.

### 2.6 Phylogenetic analysis

Forty‐two *MADS‐box* family genes of different species were selected and downloaded from UniProtKB/SwissProt database. Each gene was screened with ATG as the initiation codon and TAA, TAG, or TGA as the termination codon (Table 1). According to the downloaded CDSs of the *MADS‐box* gene family of different species, multiple sequence alignments were performed, and a phylogenetic tree was constructed to study the differences among them and the evolutionary relationship between the genes of each species. The multiple alignment of the gene sequence was completed using Clustal X (Larkin et al., 2007) software, and the default parameters were used. MEGA X (Kumar et al., 2016) software was used to calculate the best model based on maximum likelihood method and to construct the phylogenetic tree (Tamura et al., 2004), with the bootstrap value set to 1000 to verify the credibility of the evolutionary tree. The constructed evolutionary tree was made presentable using the iTOL website (https://itol.embl.de/).

**TABLE 1:** *MADS‐box* gene sequence accession numbers.

Family	Species name	ID	Gene
Asteraceae	*Helianthus annuus* L.	XM_022156766.2	AGL42
Myrtaceae	*Eucalyptus grandis* Hill ex Maiden	XM_010038958.3	AP1
Euphorbiaceae	*Hevea brasiliensis* Muell. Arg.	KY471161.1	AP1
Myrtaceae	*Syzygium oleosum* B. Hyland	XM_030586579.1	AP1
Myrtaceae	*Eucalyptus globulus* Labill.	AF306349.1	AP2L
Salicaceae	*Populus* trichocarpa	XM_02459188.1	*AGL12*
Salicaceae	*Populus euphratica* Oliv.	XM_011007131.1	*AGL12*
Juglandaceae	*Juglans regia* L.	XM_018985437.2	*AGL12*
Rosaceae	*Malus domestica* Borkh.	XM_029103631.1	Transcription factor 23 like
Lythraceae	*Punica granatum* L.	XM_031529165.1	*SVP*
Salicaceae	*Populus euphratica* Oliv.	XM_011023543.1	*SVP*
Salicaceae	*Populus* trichocarpa	XM_002310274.3	*SVP*
Lamiaceae	*Lavandula angustifolia* Mill.	MN503881.1	*SVP*
Brassicaceae	*Brassica napus* L.	JQ906720.1	*SVP*
Brassicaceae	*Brassica* juncea	JQ906716.1	*SVP*
Meliaceae	*Lansium domesticum* Corrêa	KY404063.1	*SVP*
Orchidaceae	*Paphiopedilum callosum* Stein	MT522023.1	*SVP*
Berberidaceae	*Epimedium sagittatum* Maxim	KX250266.1	*SVP*
Brassicaceae	*Arabidopsis thaliana* L.	NM_127820.4	*SVP*
Paeoniaceae	*Paeonia lactiflora* Pall.	MW039597.1	*SVP*
Iridaceae	*Crocus sativus* L.	MN015609.1	*SVP*
Salicaceae	*Populus tomentosa* Carrière	MF463462.1	*SVP*
Liliaceae	*Lilium pumilum* Redouté	MF693882.1	*SVP*
Passifloraceae	*Passiflora edulis* Sims	KY471458.1	*FUL*
Solanaceae	*Nicotiana sylvestris* Speg.	NM_001302579.1	*FUL*
Solanaceae	*Nicotiana tabacum* L.	NM_001325205.1	*FUL*
Solanaceae	*Nicotiana tomentosiformis* Goodsp.	XM_009627559.3	*FUL*
Vitaceae	*Vitis riparia* Michx.	XM_034843608.1	*FUL*
Juglandaceae	*Carya illinoinensis* K. Koch	XM_043114541.1	*FUL*
Euphorbiaceae	*Manihot esculenta* Crantz	XM_021768100.2	*FUL*
Fagaceae	*Quercus lobata* Née	XM_031097424.1	*FUL*
Rosaceae	*Rosa chinensis* Jacq.	MN119278.1	*FUL*
Fabaceae	*Glycine max* L.	NM_001254108.1	*FUL*
Anacardiaceae	*Pistacia vera* L.	XM_031426520.1	*FUL*
Malvaceae	*Gossypium hirsutum* L.	XM_041097424.1	*FUL*
Brassicaceae	*Arabidopsis thaliana* L.	NM_102396.3	*FUL*
Solanaceae	*Solanum lycopersicum* L.	NM_001374383.1	*FUL*
Malvaceae	*Theobroma cacao* L.	XM_018118461.1	*FUL*
Solanaceae	*Nicotiana* attenuata Torr. ex S. Watson	XM_019382645.1	*FUL*
Solanaceae	*Solanum pennellii* Correll	XM_015212655.2	*FUL*
Solanaceae	*Capsicum annuum* L.	NM_001324623.1	*FUL*
Solanaceae	*Solanum tuberosum* L.	XM_006345039.2	*FUL*

### 2.7 Selective pressure analysis

The codeml program in PAML 4.9 (Yang, 2007) software was used to perform selective pressure analysis on the selected sequences. Based on the self‐built evolutionary tree and the results of multiple alignment of CDSs in this study, we used the codeml program to analyze the proteins encoded by 50 CDSs, and the site models were M0, M1, M2, M3, M7, and M8. Likelihood ratio verification statistics were used to evaluate paired models, M0 versus M3, M1 versus M2, and M7 versus M8, to test whether there was a positive selection site, and a chi‐square test was performed on the results. The degree of freedom was the difference between the parameters of the two models. Finally, selection pressure analysis was performed on the selected genes according to the results.

### 2.8 Estimation of divergence time

Divergence time estimates were made for each type of gene using the MCMCTree program in PAML 4.9 (Kobayashi & Weigel, 2007) software. The MCMCTree program is based on the approximate likelihood method (Reis & Yang, 2011), which estimates the divergence time of the involved species. The age of the fossils in the self‐built tree was queried using the TimeTree website (https://www.timetree.org), where the age unit was 1 = 100 Ma. The stability of the output evolutionary tree file with divergence time was tested by Tracer V1.7.2 (Rambaut et al., 2018) software, and the parameters in PAML software were judged to be reasonable by checking whether its Ess value was >200. Finally, FigTree V1.4.4 software was used for visual analysis.

### 2.9 qRT‐PCR and expression analysis

Upper middle leaves of well‐grown plants containing flowering buds were selected at early floral stages (June) of both species to verify the differences in the expression of flowering‐related genes between the two species by qRT‐PCR. Total RNA was extracted by modified Trizol method and reverse transcribed with ReverTra Ace qPCR RT Kit (TOYOBO) (Chang et al., 2014). The sequences of the designed fluorescence quantitative primers are shown in Table S1. Using TB Green® Premix Ex Taq™II (TAKARA), we performed qRT‐PCR. qRT‐PCR analysis of each three biological samples was performed in triplicate.

## 3 RESULTS

### 3.1 Transcriptome analysis

#### 3.1.1 Analysis of orthologous gene expression

**FIGURE 1:**
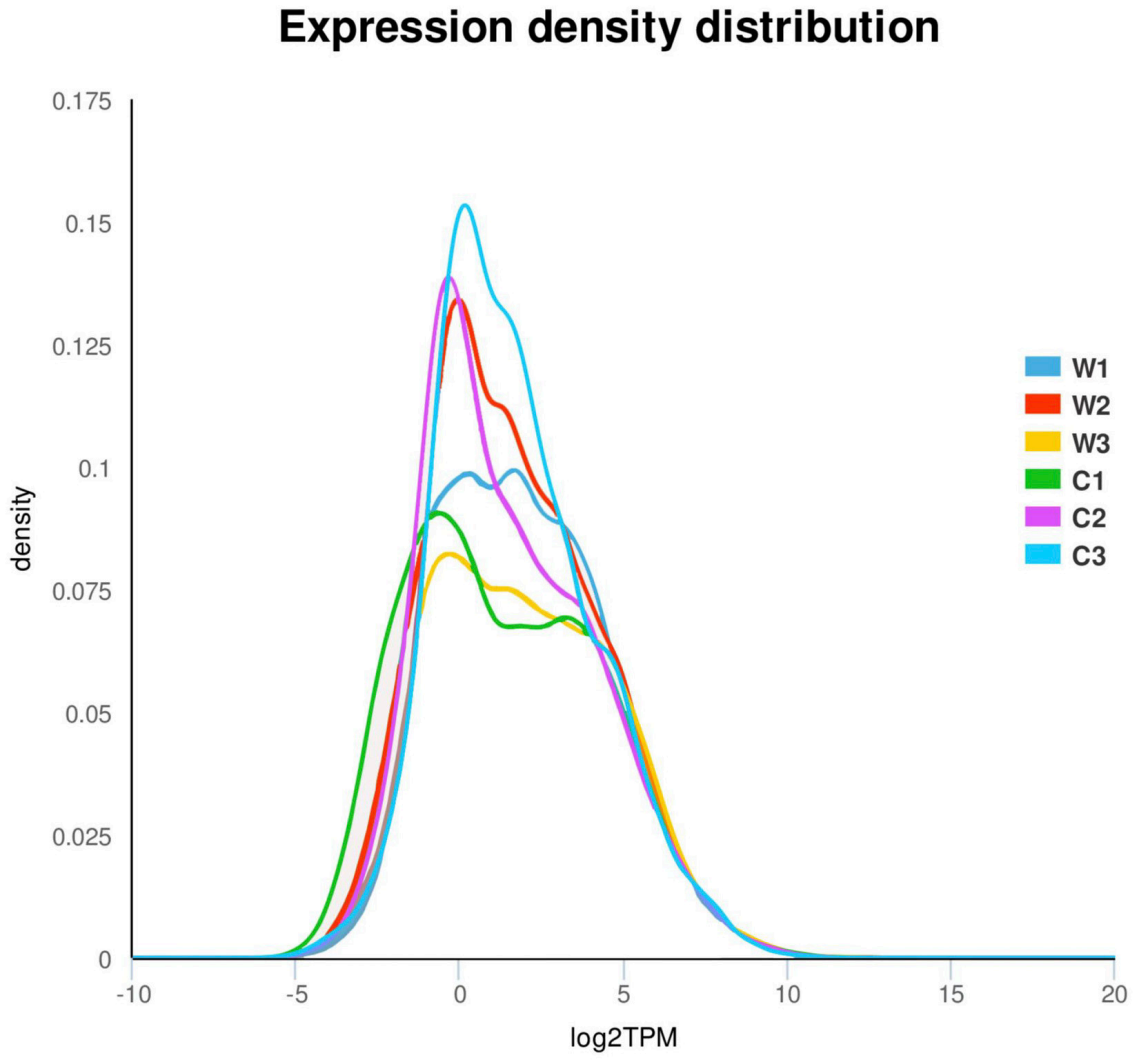
TPM scores distribution map. This figure shows the probability density distribution of the expression of all genes. C1, C2, and C3 represent three repeats of *Hypericum longistylum*. W1, W2, and W3 represent three repeats of *Hypericum attenuatum*.

The Bowtie comparison of the RSEM software was used to perform expression statistics for the results. First, we obtained the number of read counts, compared to each gene for each sample, and then performed FPKM (Fragments Per Kilobase of exon per Million fragments mapped) conversion on it to obtain the expression level of the gene. Using the CDS data of each homologous gene as the reference sequence, the abundance of CDS reflected the expression level of homologous genes. The higher the abundance is, the higher the gene expression level. The figure shows the probability density distribution of all genes (Figure 1).

#### 3.1.2 Differentially expressed gene analysis and GO, KEGG enrichment analysis

**FIGURE 2:**
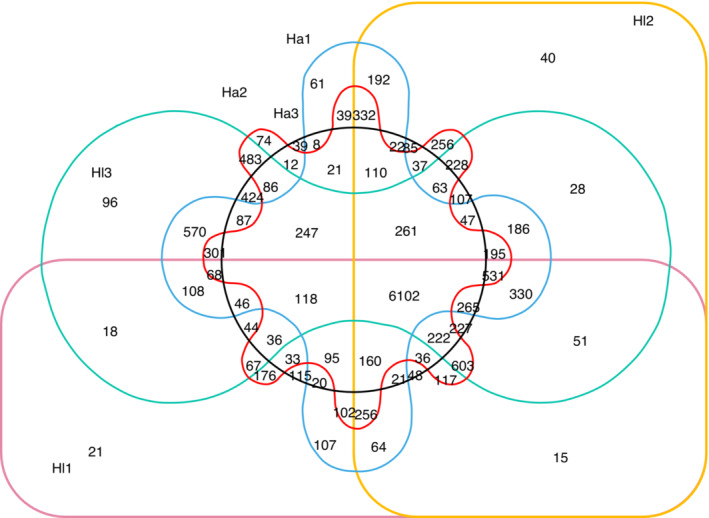
Venn of DEGs among samples. Ha1, Ha2, and Ha3 represent three repeats of *Hypericum attenuatum* and Hl1, Hl2, and Hl3 represent three repeats of *H. longistylum*.

The transcriptome data of the three samples were classified, and a total of 6044 genes were found to be present in all the six samples. In the three samples of *H. longistylum* Oliv., there were 7660 genes; a total of 6700 genes were found to be common to *H. attenuatum* Choisy (Figure 2).

**FIGURE 3:**
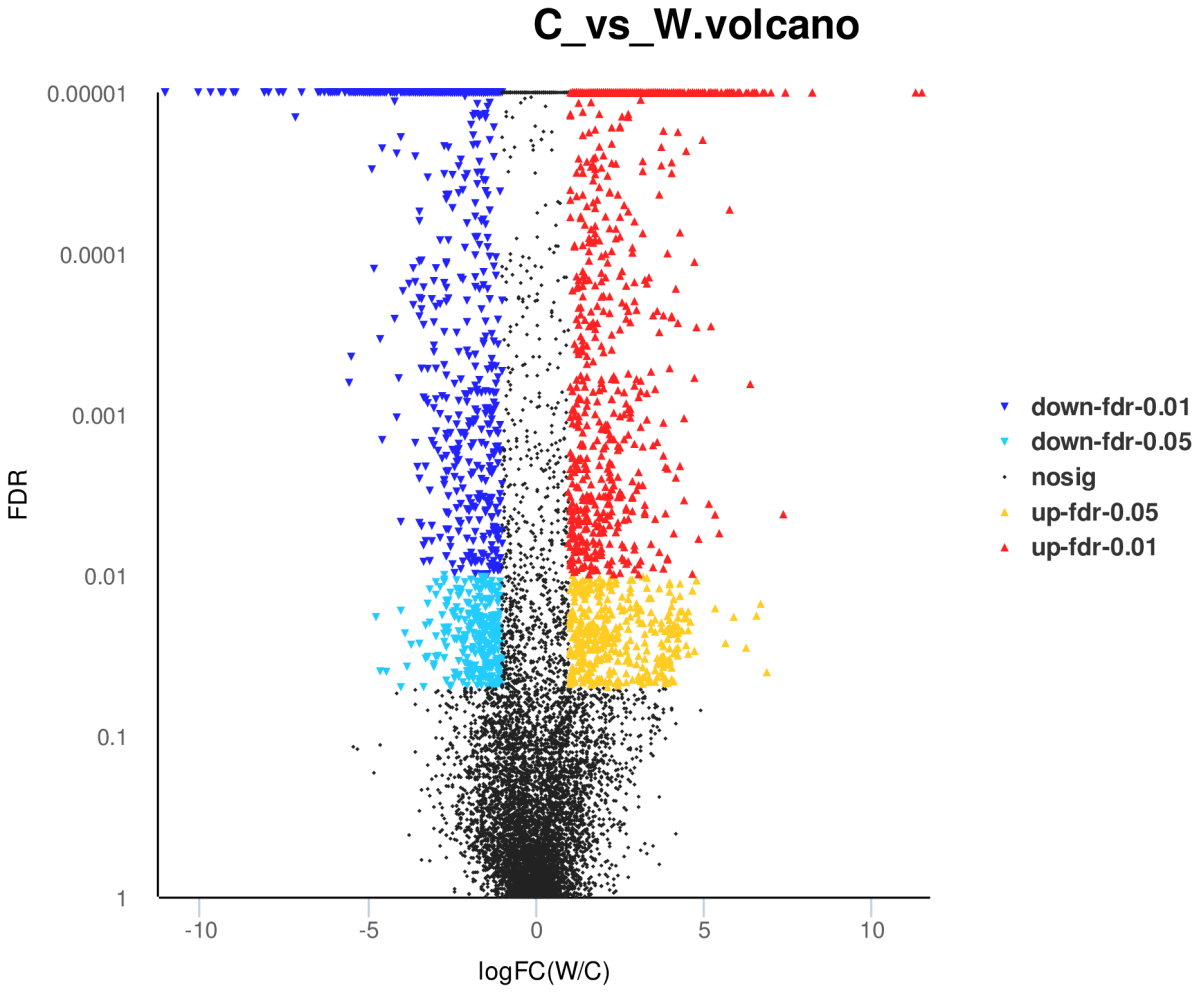
Volcano plot of DEGs. C represents *Hypericum longistylum*, and W represents *H. attenuatum*.

In this study, with FDR (false discovery rates) < 0.05 and |log_2_FC| ≥ 1 as the screening threshold, 9287 significantly DEGs were obtained, of which 4565 genes were upregulated and 4722 genes were downregulated. However, with FDR < 0.01 and |log_2_FC| ≥ 1 as the screening threshold, a total of 2301 significantly DEGs were obtained, of which 1288 were upregulated and 1013 were downregulated (Figure 3).

**FIGURE 4:**
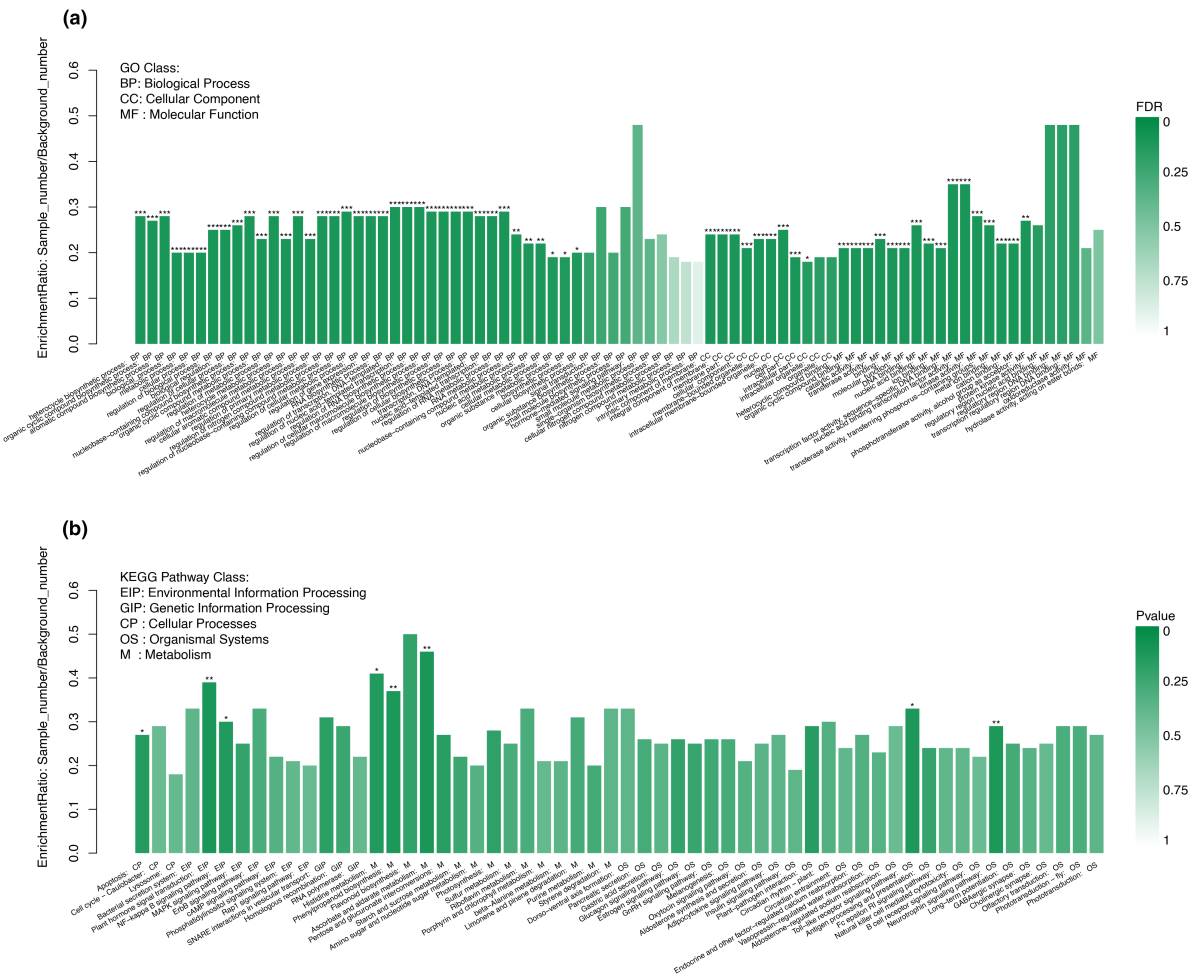
Histogram of GO (a) and KEGG (b) enrichment of DEGs. The FDR < 0.001 is marked as ***, the FDR < 0.01 is marked as **, and the FDR < 0.05 is marked as *, and the color gradient on the right represents the FDR size.

Genes could be classified according to the biological processes they participated in, the components that made up cells, and the molecular functions they performed using the GO term. The statistics of GO term annotations were performed on the DEGs in the two groups, and one of the samples was used as a control (Figure S1). In this study, GO enrichment analysis was performed on the DEGs obtained (Figure 4a). From Figure 4a, we can see that the DEGs are significantly (FDR < 0.001) enriched to 54 GO terms: In the biological process category, these differential genes were significantly enriched in 31 GO terms; In the cellularity category, eight GO terms were significantly enriched; In the molecular function category, 15 GO terms were significantly enriched.

As can be seen from KEGG enrichment analysis (Figure 4b), all DEGs were significantly enriched into eight pathways, among which four were highly significantly enriched (FDR < 0.01), respectively: plant hormone signal transduction in environmental information processing and phenylpropanoid in metabolism biosynthesis, ascorbate and aldarate metabolism, and neurotrophin signaling pathway in organismal systems.

### 3.2 Prediction of *MADS‐box* gene domains and conserved motifs

**FIGURE 5:**
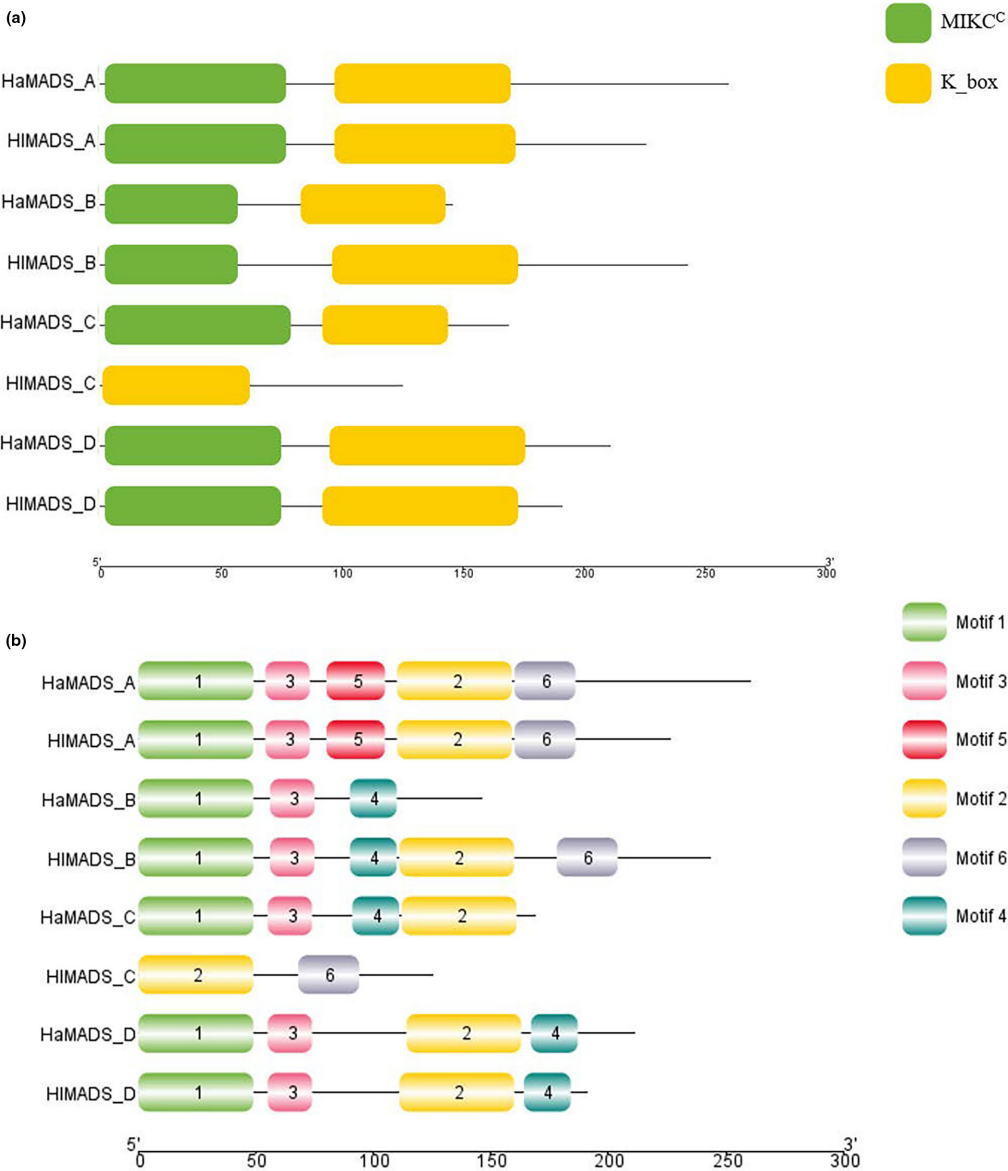
Conserved domains (a) and motif (b) of differentially expressed *MADS‐box* genes in two species.

Among the DEGs, we enriched a total of nine pairs of *MADS‐box* family genes, among which four pairs were significant (data not provided). Each gene corresponded to a sequence in *H. longistylum* and *H. attenuatum*, respectively. We made domains and conserved motifs prediction of the amino acid sequences of these four pairs of genes (Figure 5). The detailed sequences are shown in Table 2. Through domain's prediction results, we found that these DEGs all contained a K region, and only HlMADS_C gene did not contain MEF2_like region. According to the motifs distribution (Figure 5b), except HlMADS_C and HaMADS_B genes, the other genes contain at least four different motifs, and only A‐class genes contain motif 5, while HlMADS_C only contains motif 2 and motif 6.

**TABLE 2:** The detailed sequences of the domain and motif.

Domain	Sequence
MADS_MEF2_like	MGRGRVQLKKIENKINRQVTFSKRRSGLLKKAHEISVLCDAEVGLIIFSSKGKLFEYATGSCMERILERYERYSYAER
K‐box	ILSKEVAKKSHQLRQMRGEELQGFTLEELQQLENSLENGLARVIEKKGEKIMKEISELQKKGMQLMEENVRLRQ
Motif 1	MGRGKVZJKRIENPTSRQVTFSKRRSGLLKKAKEJSVLCDAEVALIVFSS
Motif 2	MKGEELDGLSLKELQQLEQQLESALKHIRTRKGDLMYKEISELQKKEGAL
Motif 3	FEATDSSMDEILERYEKHSY
Motif 4	WELEQAKLESRAEILQRSPRN
Motif 5	QPSLELQLVEDPNRSILSEEVAKKSH
Motif 6	MEENARLKQQASJLNGPAVIATPPPQP

### 3.3 Multiple sequence alignment and phylogeny analysis

**FIGURE 6:**
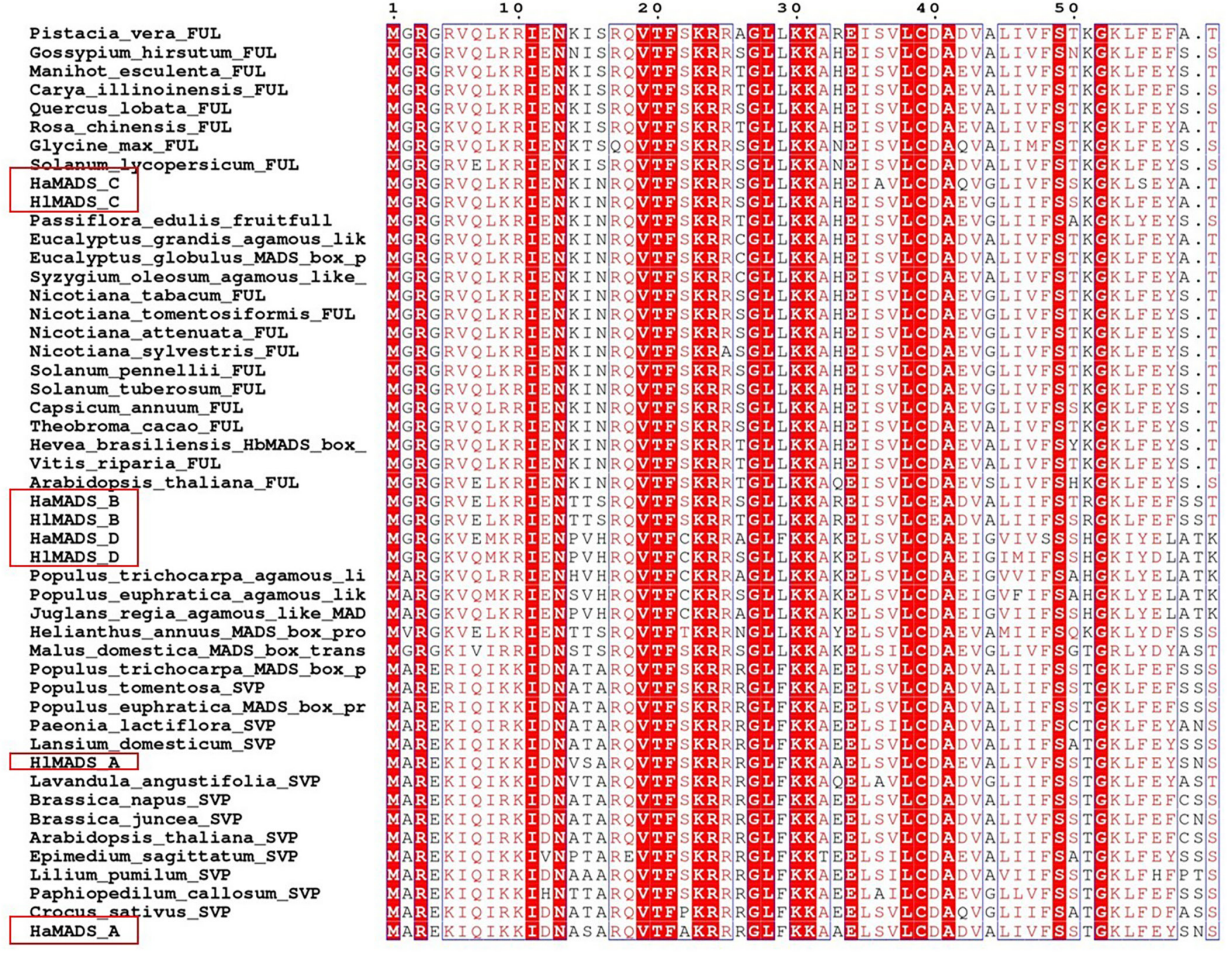
Partial multiple sequence alignment results of *MADS‐box* genes.

Clustal X 2.1 software was used to perform multiple sequence alignment on the screened hypericum gene sequences and the CDSs of the *MADS‐box* gene family of the selected 42 other species (Figure 6). From the results of the multiple sequence alignment, we found that the A group genes have a relatively high homology to the *SVP* (SHORT VEGETATIVE PHASE) gene, and the B group genes and C group genes have high homology to the *FUL* (*FRUITFULL*) gene, while the D group gene has a high homology with the AGL12 (AGAMOUS LIKE 12) gene of the two hypericums.

**FIGURE 7:**
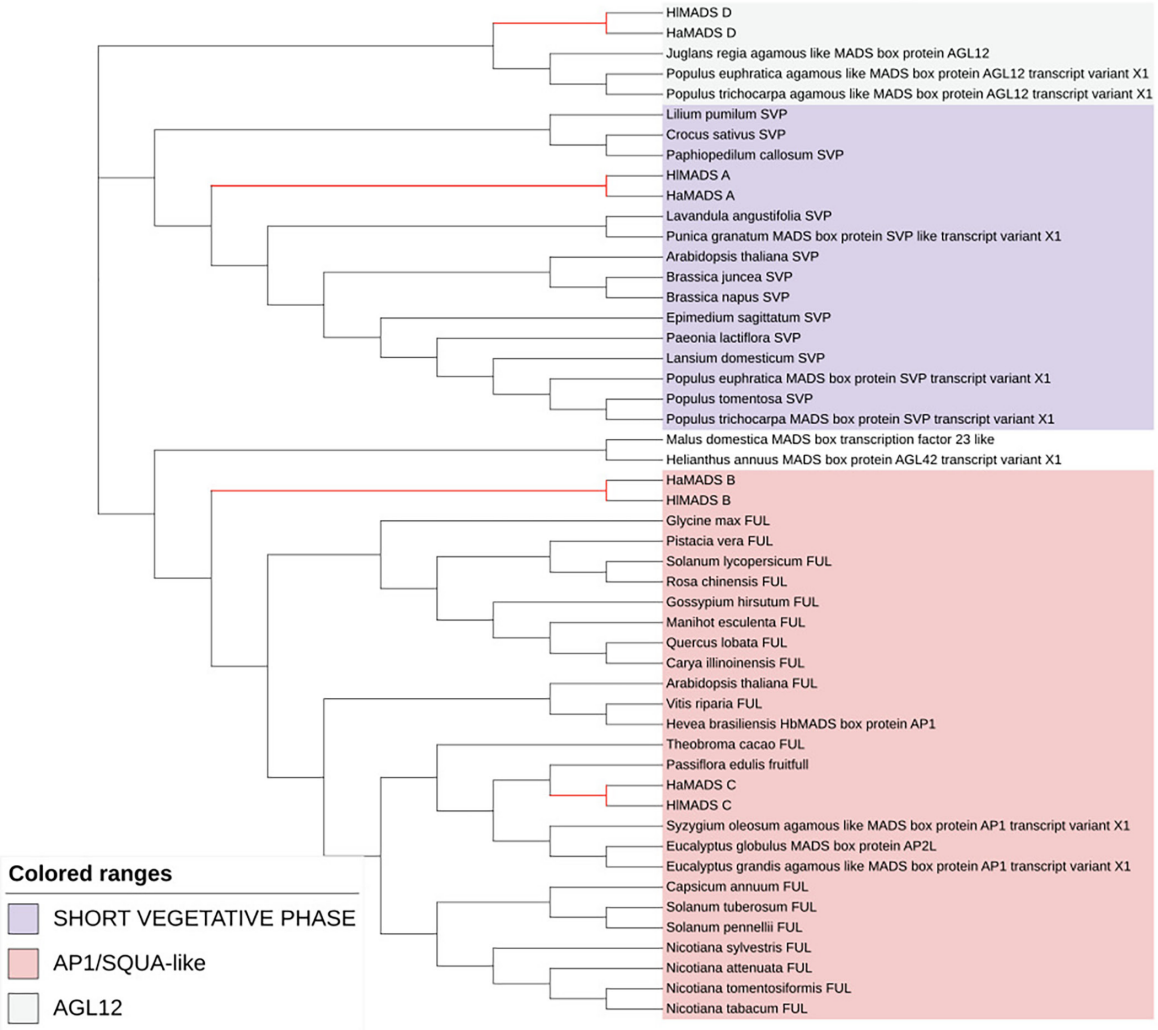
*MADS‐box* gene family phylogenetic tree. The red branches in the figure represent the gene sequences in this study, and the genes in the same background color are grouped into one category and are divided into three categories.

The sequence after multiple sequence alignment was used to calculate the best model of the paper mulberry tree using MEGA X software. The results showed that the best model was GTR + G + I, which could be used to build a phylogenetic tree and beautify it (Figure 7). The phylogenetic tree is output as an NWK file. Through the evolutionary tree, these 50 *MADS‐box* genes can be divided into AGL12, SHORT VEGETATIVE PHASE, and AP1/SQUA‐like. The red branch line indicates the *MADS‐box* family genes screened from the transcriptome of this study. D group genes are clustered with the AGL12 gene in the *MADS‐box* family, the A group genes are clustered with the *SVP* gene in the *MADS‐box* family, and the B genes are clustered with the C genes. In Group B and Group C, which are grouped together, the evolutionary relationship between Group C genes and *Passiflora edulis* is closer, while the evolutionary relationship between Group B genes and the branch as a whole is farther away. Therefore, the genes in Group A are *SVP* genes, the genes in Groups B and C are *FUL* genes, and the genes in Group D are AGL12 genes. From the results of this evolutionary tree, the differentially expressed *MADS‐box* gene family in Changbai Mountain hypericum can be divided into these three branches.

### 3.4 Select pressure analysis

The *MADS‐box* gene family of four pairs of homologous genes encoding proteins were analyzed by different models. The four pairs of encoded proteins were analyzed by the codeml program to obtain the relevant parameters of different Models M0 (single ratio), M1 (near neutral), M2 (positive selection), M3 (discrete), M7 (beta), and M8 (beta&ω) (Table 3). There is a correspondence between the models; M0, M1, and M7 correspond to M2, M3, and M8, respectively. Subsequent LRT (likelihood ratio test) statistics and chi‐square tests were used to determine whether the model was significant. Table 4 shows that the M2, M3, and M8 models of the *MADS‐box* gene family are superior to the M1, M0, and M7 models and reach a very significant level (*p* < .05). Table 2 shows that there were values of *ω* > 1 in M2, M3, and M8; that is, positive selection exists. Bayesian methods based on Bayesian experience further consider M2 and M8. The M2 model calculated seven positive selection sites, which are 266 A, 267 F, 284 P, 304 K, 339 Y, 348 I, and 349 F. The M8 model calculated six positive selection sites, which are 267 F, 284 P, 304 K, 339 Y, 348 I, and 349 F.

**TABLE 3:** Adaptive evolution analysis of *MADS‐box* proteins.

Models	np	InL	Parameters			
M0	99	−14876.486671	*w*	0.47725		
M1	100	−14355.495395	*p*	0.39211	0.60789	
			*w*	0.14167	1	
M2	102	−14330.104183	*p*	0.35158	0.492949	0.15593
			*w*	0.13294	1	2.02245
M3	103	−14260.996433	*p*	0.20006	0.28834	0.5116
			*w*	0.03487	0.32537	1.02552
M7	100	−14255.617905	*p* = 0.47225	*q* = 0.36033		
M8	102	−14237.260187	*p*0 = 0.84086	*p* = 0.50261	*q* = 0.52013	
			(*p*1 = 0.15914)	*w* = 1.65345		

**TABLE 4:** *MADS‐box* protein positive selection site.

Models	df	LRT	*p*‐value	Positive selection site
M0 VSM3	4	1230.980476^**^	3.06E−265	7
M1 VSM2	2	50.782424^**^	9.39E−12	Not detected
M7 VSM8	2	36.715436^**^	1.06E−08	6

** represents significance p < .01.

### 3.5 Analysis of divergence time

**FIGURE 8:**
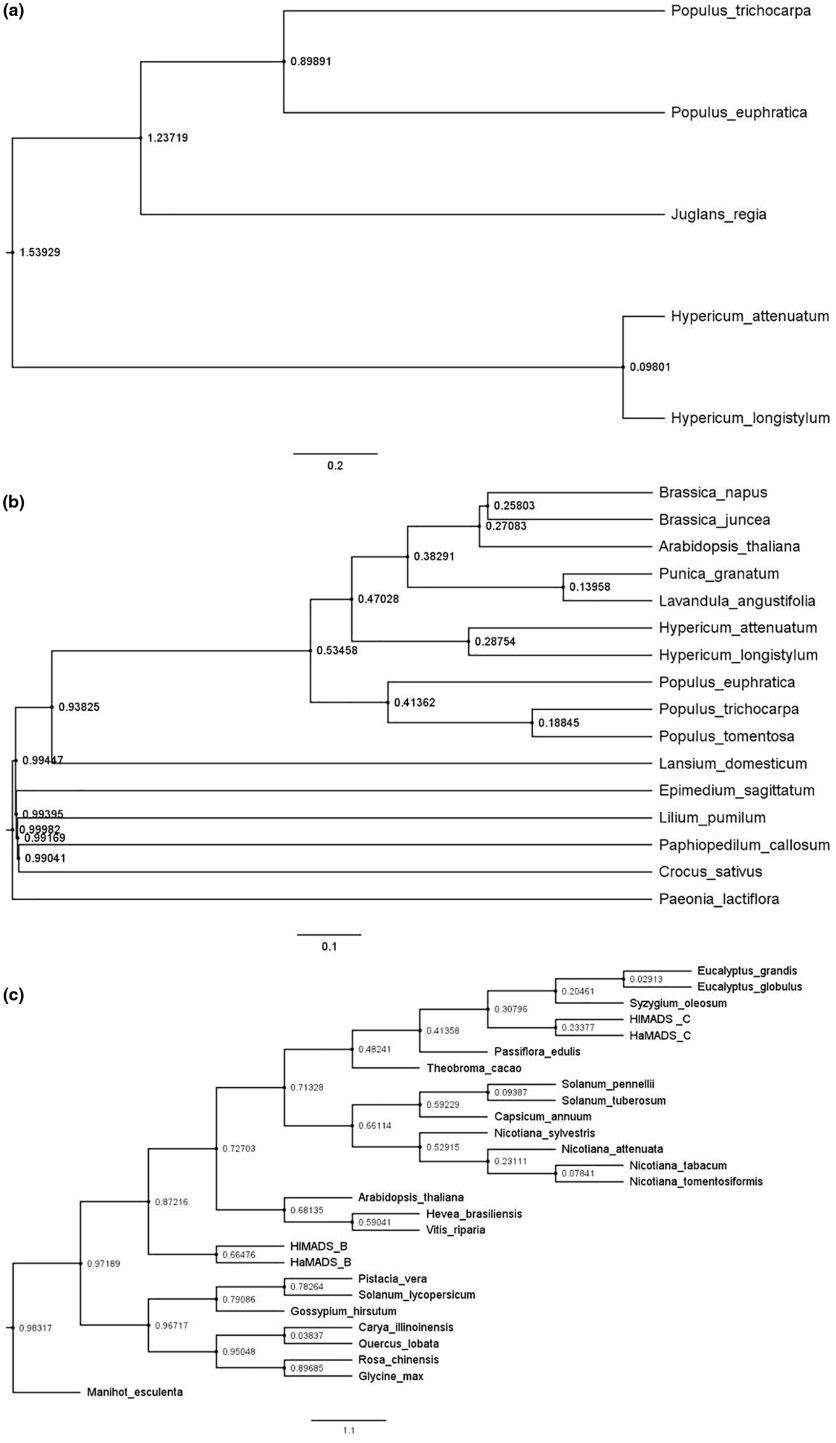
The divergence time of *AGL12* (a), *SVP* (b), and *FUL* (c).

The MCMCTree program was used to estimate the divergence time of the three types of genes. Tracer v1.7.2 software found that the Ess values were all >200, indicating that the parameters were reasonable. Finally, the divergence time of each gene was obtained separately, and a tree was constructed (Figure 8). The numbers in the figure represent the divergence time. For the AGL12 gene, *H. longistylum* and *H. attenuatum* were separated in the Tortonian Stage (approximately 11.61 Ma–7.25 Ma) of the Miocene, at approximately 9.801 Ma. In the evolutionary tree of the *SVP* gene, *H. longistylum* separated from *H. attenuatum* in the Rupelian Stage (approximately 33.9–28.4 Ma) of the Oligocene, was at approximately 28.754 Ma. Finally, the *FUL* gene was separated into the Chattian Stage (approximately 28.4–23.03 Ma) of the Oligocene and Maastrichtian Stage (approximately 70.6–65.5 Ma) of the Late Cretaceous at approximately 23.377 and 66.476 Ma, respectively.

### 3.6 Quantitative real‐time fluorescence analysis

**FIGURE 9:**
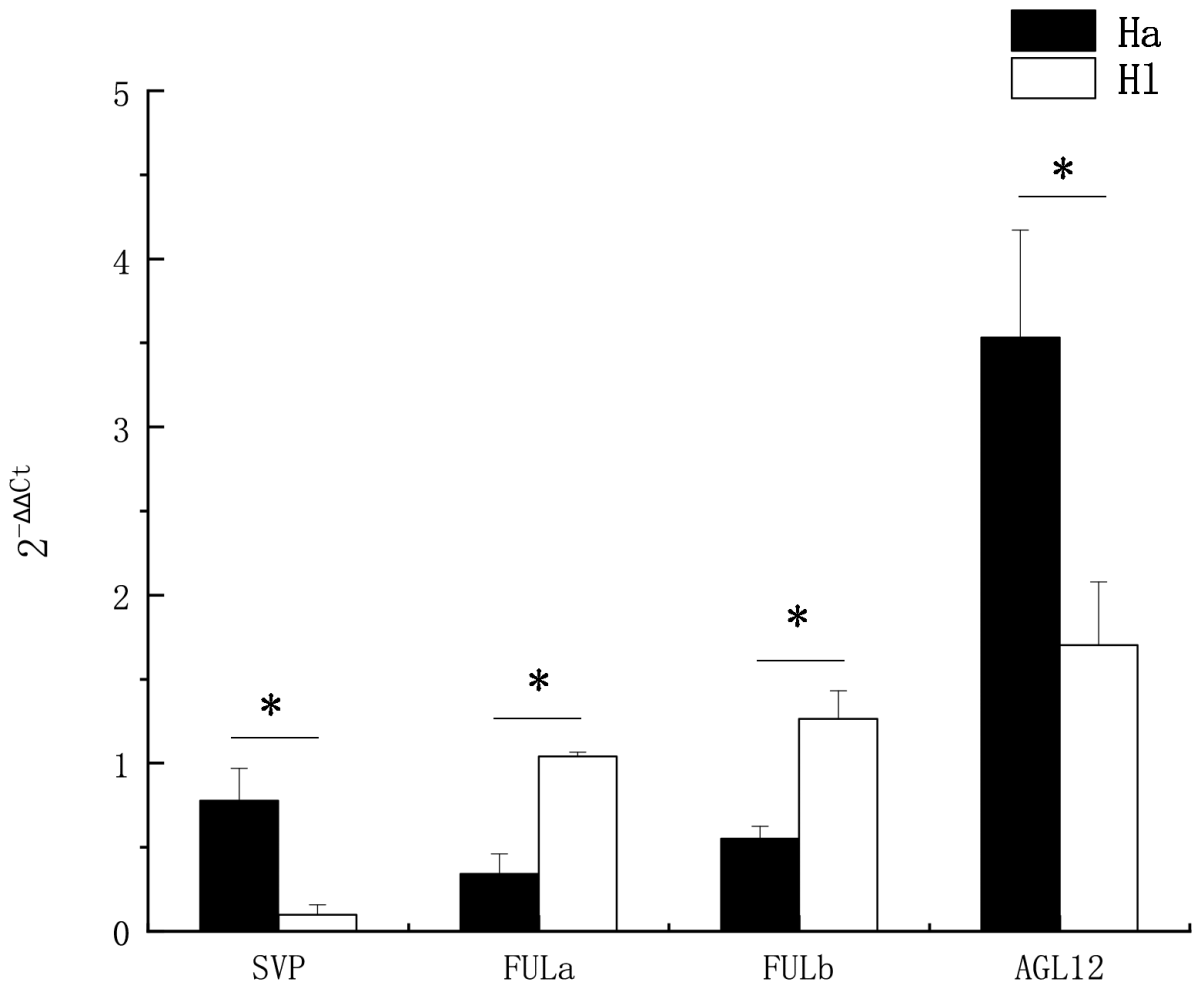
Expression analysis graph. The ordinate in the figure is the value of $2^{\left(-∆∆C_{t}\right)}$, and the higher the value, the higher the gene expression. Ha means *Hypericum attenuatum*, and Hl means *Hypericum longistylum*.

To further determine the relative expression levels of *MADS‐box* gene in the two plants, quantitative real‐time PCR was performed, and the results are shown in Figure 9. As can be seen from the figure, the expression levels of the *SVP* and AGL12 in *H. attenuatum* were higher than those in *H. longistylum*, and reached a significant level (*p* < .05). And the relative expression levels of the two *FUL* genes in *H. longistylum* were higher than those in *H. attenuatum*, and reached a significant level (*p* < .05).

## 4 DISCUSSION

In this study, comparative transcriptome sequencing was used to analyze two species of Hypericum in Changbai Mountains. The results showed that 9287 genes (including 4565 upregulated genes and 4722 downregulated genes) with significant differences were obtained (FDR < 0.05). Among them, we detected 6044 DEGs in all six samples. However, 2301 DEGs (1288 upregulated genes and 1013 downregulated genes) reached the highly significant level (FDR < 0.01). In the subsequent GO enrichment analysis, it was found that these significantly different genes (FDR < 0.05) were mainly enriched into 54 GO terms. KEGG enrichment analysis found that the genes with significant differences were mainly enriched in plant hormone signal transduction, phenylpropanoid biosynthesis, ascorbate and aldarate metabolism, and neurotrophin signaling pathway.

*MADS‐box* family genes have been cloned and verified in many plants, and the functions of *MADS‐box* family genes are closely related to the flowering process of plants (Bowman et al., 1991; Thessen et al., 2000; Wang, Song, et al., 2022). As a family gene, *MADS‐box* has high conservation. According to the results of domain prediction analysis, all the gene sequences involved in this study were detected to contain a K region. Kerstin et al. found that the K region is often used to distinguish type I and type II *MADS* genes (Kerstin et al., 2005; Zhao et al., 2021). Therefore, it can be concluded that type II *MADS‐box* genes are significantly different among different species of Hypericum in Changbai Mountains. Combined with motif prediction analysis, Motif 1 and Motif 3 formed the MEF2_like domain, while Motif 2, 4, and 6 formed the K domain. Some studies have shown that the type of motif is related to the type of gene function (Liu et al., 2022; Won et al., 2021). Wang et al. showed that conserved motifs in the tomato MADS‐box family play an important role in population‐specific functions. However, there is a high degree of structural variation between the different groups. This could provide additional clues to the evolutionary relationships of the tomato MADS‐box family (Wang et al., 2019). Shen et al. studied differences in the B protein motif to better understand the functional evolution of this protein (Shen et al., 2021). Therefore, the presence of motif 5 only in group A genes may be the reason why the function of this group of genes is different from the rest of the genes.

Multiple sequence alignment and phylogenetic analysis showed that group A genes were *SVP* genes, group B and C genes were *FUL* genes, and group D genes were *AGL12* genes. Among them, group C genes are closely related to *P. edulis*, while group B genes, although classified as *FUL* genes, are far from each other. The results of selection evolution analysis showed that there was positive selection for *MADS‐box* genes differentially expressed in the two Hypericum species in Changbai Mountain area, and M2 and M8 respectively had six and seven positive selection sites. This means that these genes are being influenced by Darwinian selection evolution (Yang, 2006). Hypericum species in Changbai Mountains are also in the environment of adaptive protein evolution.

According to the results of divergence time, it can be seen that the flowering genes of *H. longistylum* and *H. attenuatum* were separated in the Cenozoic period. The most striking feature of this period is the high level of mammalian and angiosperms (Jiang et al., 2012; Santiago et al., 2020). The Oligocene period covered in this study was dominated by the evolution and spread of modern types of flowering plants, and large‐scale temperature changes occurred during this period (Ge et al., 2006; Jia et al., 2004; Ma & Gao, 2003; Zachos et al., 2001). This makes it possible for plants to evolve different coping strategies to adapt to such drastic changes, and to carry this possibility into their genes. This may be the reason why *SVP* and *FUL* genes, which regulate flowering and flower size, are separated. Although the exact differentiation time of this species is not known, studies on the same genus *H. perforatum* show that the differentiation time of this genus separated from *Linum* at about 100 Ma, which was in the transition stage from the early Cretaceous to the late Cretaceous (Wen et al., 2021). The divergence times in other species of the genus showed that there was a great deal of separation between 24.4 and 42.9 Ma (Kumar et al., 2017; Meseguer et al., 2013). Therefore, it is reasonable to speculate that *SVP* gene (group A) and *FUL* gene (group C) were separated during the differentiation of these two species. It was found that Hypericum underwent two genome‐wide replication events at 9.16 and 57.96 Ma, respectively (Wen et al., 2021). Therefore, we speculate that AGL12 gene (group D) was isolated during a genome‐wide replication event about 9.16 Ma. However, *FUL* genes in group B were isolated during a genome‐wide replication event about 57.96 Ma.

The analysis of DEGs in different species of the same genus has been involved in many species studies (Guo et al., 2021; Jiang et al., 2021; Zhu, 2020). Studies had shown that high expression of SVP resulted in smaller flowers and also inhibited the flowering process in most dicotyledonous plants (Jaudal et al., 2014). In addition to this, *AGL12* suppressed the process of flowering transformation in plants, while *FUL* promoted the formation of inflorescence meristematic tissue in plants at an early stage of flower development (Kempin et al., 1995; Li et al., 2008; Tapia‐Lopez et al., 2008). The results of some other studies showed that both *SVP* and *AGL12* inhibited flowering transition, while *FUL* promoted the formation of inflorescence meristem at the early stage of flower development (Kempin et al., 1995; Li et al., 2008; Tapia‐Lopez et al., 2008). It can be seen from the results of this study that the expression levels of *SVP* and AGL12 in *H. attenuatum* were higher than those in *H. longistylum*, while the expression levels of *FUL* were lower than those in *H. longistylum*. Therefore, in *H. attenuatum*, the flowering transition process and flower meristem formation process development was inhibited at the early stage of flowering time, which was consistent with the phenomenon that the flowering stage of *H. attenuatum* was later than that of *H. longistylum*. Some studies had pointed out that the expression and function of the same genes may differ in different species, for example, the presence of high SVP expression in both mango and soybean was found to promote plant flowering (Mo et al., 2021; Woods et al., 2016). Therefore, the specific functions of the MADS‐box family in Hypericum remain to be further investigated subsequently.

## 5 CONCLUSION

In this study, a total of 9287 DEGs were detected in two Hypericum species in Changbai Mountain, and 6044 genes were expressed in both species. Through subsequent KEGG pathway analysis, it was found that these DEGs were mainly enriched into four pathways. Four pairs of differentially expressed *MADS* genes related to flowering were screened out in this study, and it was found that they were influenced by Darwinian selection evolution, and their growth environment was suitable for such selection. The divergence time analysis showed that the divergence of type II *MADS* genes between the two species may be related to environmental changes and their own genome replication events. The relative expression analysis showed that the high expression of *SVP* and *AGL12*, which inhibited flowering, and the low expression of *FUL*, which promoted flower development, might be internal factors contributing to the later flowering of *H. attenuatum* than *H. longistylum*.

The purpose of this study was only to investigate the differences between the MADS‐box flowering genes of the two species, and the specific functions of their genes were not verified. Therefore, further studies are needed.

## AUTHOR CONTRIBUTIONS

**Xia Yunrui:** Writing – original draft (equal). **Song Rui:** Data curation (equal). **Yang Xing:** Software (equal). **Zhao Zhe:** Visualization (equal). **Zhang Keqin:** Methodology (equal). **Zhang Nanyi:** Writing – review and editing (equal).

## Supporting information


Figure S1

Table S1

## Data Availability

Nucleotide sequences: GenBank accession numbers for my nucleotide sequences ON263567‐263574 in NCBI (https://www.ncbi.nlm.nih.gov/). Find other nucleotide sequences uploaded as online and their accession numbers are shown in Table 2.
