# Supplementary figures and images for: Comparative transcriptomic analysis reveals differences in MADS‐box genes of different hypericum in Changbai Mountains
Source: Ecol Evol. 2023 Jun 13;13(6):e10196. doi: 10.1002/ece3.10196 (PMC10261973; doi:10.1002/ece3.10196)

Biological Process

Cellular Component

Molecular Function

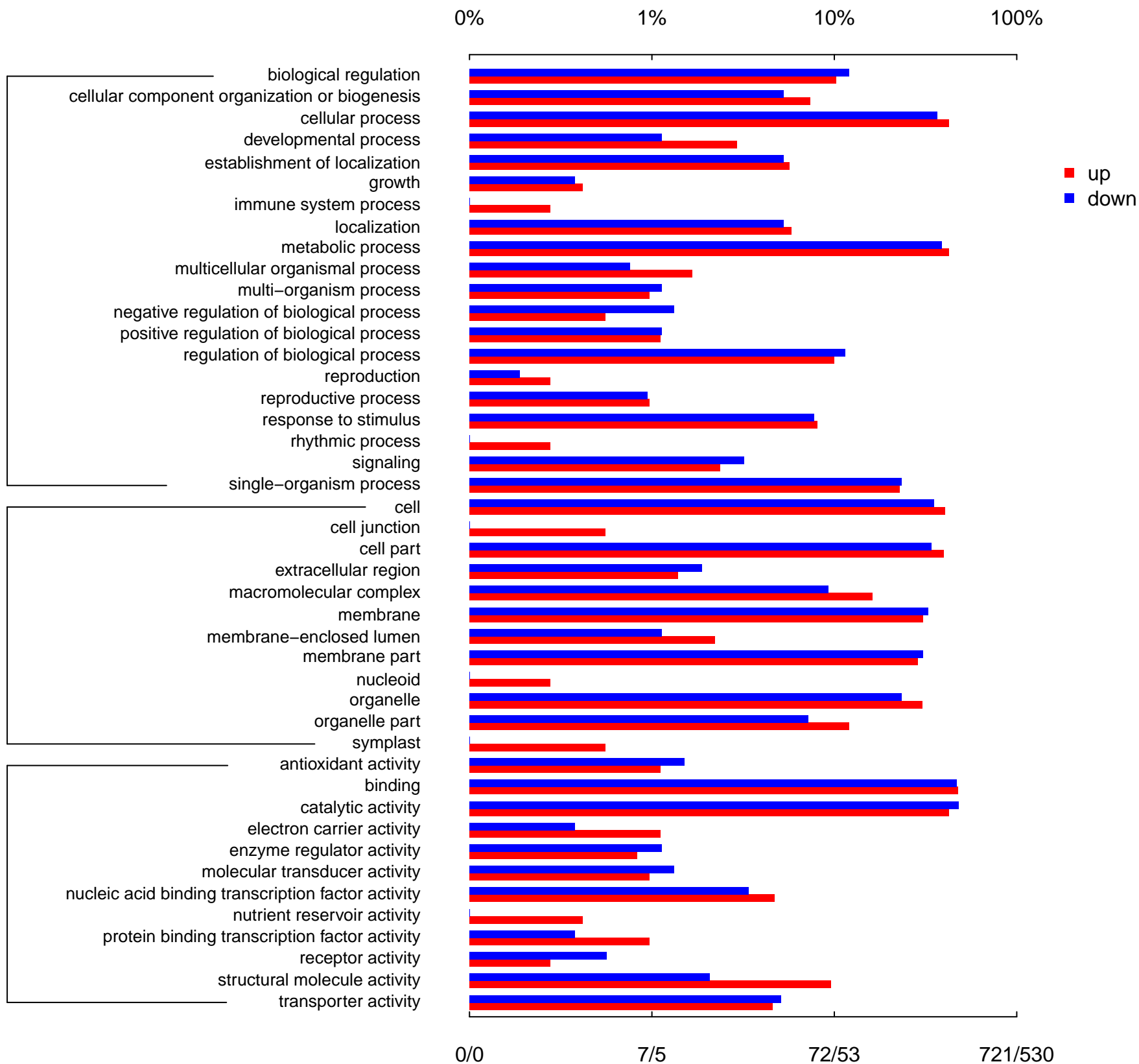

Number of genes ( Up/Down )

Supplement: Supplementary file 1 — Figure S1 [file ECE3-13-e10196-s002.pdf]
